# Novel γ-2-Herpesvirus of the *Rhadinovirus* 2 Lineage in Gibbons

**DOI:** 10.3201/eid1005.030964

**Published:** 2004-05

**Authors:** Renan Duprez, Emmanuelle Boulanger, Yannick Roman, Antoine Gessain

**Affiliations:** * Institut Pasteur, Paris, France; † Museum National d’Histoire Naturelle, Clères, France

**Keywords:** Herpesviruses, Rhadinoviruses, gibbon, Asian apes, consensus-degenerate primers, phylogeny

## Abstract

We obtained 475 nucleotides of the DNA polymerase gene of a novel human herpesvirus 8 homolog sequence in a gibbon. The finding of this new gibbon virus, which clusters with a related chimpanzee virus in the rhadinovirus 2 genogroup, suggests the existence of a novel γ-2*-*herpesvirus in humans.

Among Gammaherpesvirinae, Epstein-Barr virus (EBV) and Kaposi sarcoma–associated herpesvirus or human herpesvirus 8 (KSHV/HHV-8) represent human prototypes of the *Lymphocryptovirus* (γ-1-herpesvirus) and *Rhadinovirus* (γ-2-herpesvirus) genuses, respectively (*1*). Both viruses play an important role in human multistep carcinogenesis, especially in immunodeficient patients. Indeed, Kaposi sarcoma and EBV-associated Burkitt lymphoma currently represent the most frequent cancers in some geographic areas, especially in Africa.

Several γ-1-herpesviruses have been isolated from nonhuman primates, especially African and Asian apes, since the 1970s (*2*,*3*). However, most of the sequences of these viruses, which allow phylogenetic studies, have only been recently obtained (*4*).

Rhadinoviruses (or γ-2*-*herpesviruses) have also been found in many animal species including New and Old World primates. Among the latter, recent comparison and phylogenetic analyses of all available sequences support the existence of two distinct genogroups called RV1 and RV2 for Rhadinovirus genogroups 1 and 2 (*5*–*9*).

Among African great apes, four γ-2-herpesviruses have recently been discovered. These are PanRHV1a/PtRV1 (Pan rhadinovirus 1a/*Pan troglodytes* rhadinovirus 1), PanRHV1b (Pan rhadinovirus 1b) and GorRHV1 (gorilla rhadinovirus 1) from chimpanzees and gorillas respectively, in the RV1 group and PanRHV2 (Pan rhadinovirus 2) from chimpanzees in the RV2 group (*7*,*10*,*11*). By contrast, γ-2-herpesvirus has never been found, so far, in Asian apes. The goal of this study was therefore to search for γ-2-herpesviruses in Asian apes.

To look for KSHV-related viruses, we first performed a serologic analysis. Plasma of 38 captive Asian apes, including 30 orangutans (25 *Pongo pygmaeus pygmaeus* and 5 *P. p. abelii*) and 8 gibbons (including 1 *Hylobates gabriellae* and 7 *H. leucogenys*), originating from different zoos and primate centers mainly from France, were tested by two different immunofluorescence assays, as previously described (*12*,*13*). Briefly, the first method, which uses KSHV-infected human KS1 cells as source of antigens, allows mainly the detection of antibodies directed against lytic KSHV antigens, while the second, using KSHV-infected BC3 cells, allows only for the detection of antibodies directed against the latent nuclear antigen. Results demonstrated a clear antilytic cross-seroreactivity in plasma samples from 10 orangutans and 3 gibbons with titers from 1/20 to 1/160, while seroreactivity against the latent nuclear antigen was more rarely detected (7 orangutans and 2 gibbons), mostly with very faint patterns.

We then performed a polymerase chain reaction (PCR)-based study using high molecular weight DNA extracted either from peripheral blood mononuclear cells or from buffy-coat of 35 of these Asian great apes (8 gibbons and 27 orangutans for which DNA was available). We attempted to amplify a small fragment of the highly conserved herpesvirus DNA polymerase gene by heminested PCR, using previously described consensus-degenerate primers (*7*,*8*,*14*). Among the 35 DNA samples, 18 scored positive (5 gibbons and 13 orangutans) on the ethidium bromide gel, with a band of the expected size (237 bp), corresponding to the KSHV-positive control. Purifying, cloning, and sequencing of these products indicated the presence, in 8 orangutans and 4 gibbons, of two species-specific lymphocryptovirus sequences, that were slightly different from each other but very closely related to EBV, as recently reported (*4*). However, in 1 *H. leucogenys* (gibbon 7*)*, database searches, using BLAST web server (available from: http://wwww.ncbi.nlm.nih.gov/blast/Blast.cgi), indicated a novel γ-herpesviral DNA polymerase sequence that was closely related to the *Rhadinovirus* genus strains. No other novel herpesviral sequence was detected in orangutans or gibbons.

Using the same PCR approach with a specific reverse primer for the second PCR (HyloRHVas: CAT CGT GCG TCC CTG CAG CG), we amplified, from the DNA sample of gibbon 7, a second overlapping fragment, resulting in a 475-bp final fragment of a new herpesviral DNA polymerase gene (GenBank accession no. AY465375). Nucleotide comparison of a 451-bp fragment, corresponding to the best alignment of all the γ-herpesviruses available sequences, indicated that the novel gibbon rhadinovirus sequence was more closely related to the corresponding sequences of the RV2 genogroup viruses (76%, 73%, and 71% of nucleotide identity with ChRV2 (Chlorocebus rhadinovirus 2), MndRHV2 (Mandrillus rhadinovirus 2) and PanRHV2, respectively), than to the corresponding sequences of the RV1 genogroup viruses (70%, 69%, and 63% of nucleotide identity with KSHV, PanRHV1a, and PanRHV1b fragments, respectively). Similar results were obtained when the comparison was done on the amino acid sequences.

Phylogenetic analyses, by using two different methods (neighbor joining and DNA maximum parsimony), were performed with two different sets of sequences. The first set comprises all available primate γ-herpesvirus DNA polymerase gene sequences. The second set comprises most of the available corresponding herpesvirus sequences, including the γ-2-herpesviruses originating from nonprimates species. Nearly identical tree topologies were obtained for the two phylogenetic methods when the analyses were restricted to the primate sequences (Figure 1; data not shown). The addition of the γ-2- nonprimate herpesviruses (Figure 2) modified the positioning of some sequences within the γ-2 herpesviruses and some bootstrap values as compared to the analysis restricted to primate viruses. However, all analyses clearly localized, with bootstrap values from 83% to 85%, the novel gibbon viral sequence (HyloRHV2) within the *Rhadinovirus* genus in the RV2 genogroup (Figures 1 and 2; data not shown). All together, these studies as well as previous works, demonstrate the existence, among the primate γ-2-herpesviruses, of three distinct separate lineages, as seen on Figure 1. The first lineage corresponds to the New World group, including rhadinoviruses of spider and squirrel monkeys. The second lineage, the RV1 group, comprises the rhadinoviruses of Old World primates, including those of humans, chimpanzees, gorillas, African green monkeys, mandrills, and macaques. The third lineage, which corresponds to the RV2 group, also contains rhadinoviruses of Old World nonhuman primates (chimpanzees, African green monkeys, macaques, baboons, mandrills, and our novel gibbon HyloRHV2). The novel HyloRHV2 sequence clearly localized with the PanRHV2 strain, a recently reported strain from chimpanzees, and formed a distinct genogroup within the RV2 clade, supported by a bootstrap value of 85% (Figure 1). These two sequences, which branch off alone in the RV2 genogroup, independently of all other Old World monkey viral strains, may represent the prototype strains of a great apes lineage similar to that found in the RV1 genogroup. Based on the established view that herpesviruses have diverged from a common ancestor, in a manner mediating cospeciation of herpesviruses with their host species through latent infection, such findings reinforce the hypothesis of a putative RV2-related herpesvirus in humans.

**Figure 1:**
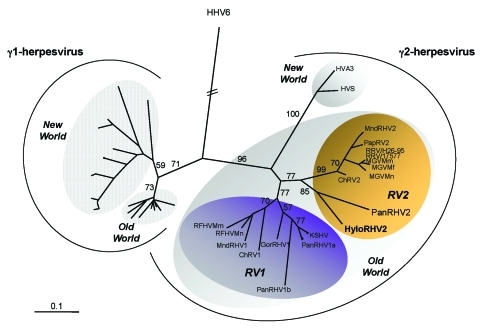
Neighbor-joining protein distance tree for the best 130 amino acids of DNA polymerase residues alignment. DNA sequences (primers DFASA and GDTD1B) (8) were first translated, then aligned by using ClustalX (nonphylogeneticaly informative gaps were manually removed) and analyzed by using the PROTDIST and NEIGHBOR programs in PHYLIP (available from: http://evolution.genetics.washington.edu/phylip.html). Horizontal branch lengths are drawn to scale, with the bar indicating 0.1 amino acid replacements per site. Numbers in each internal branch indicate the percentage of bootstrap samples (of 1,000) in which the cluster is supported (SEQBOOT). Previously published sequences included and their accession numbers are as follows: HyloRHV2 (AY465375); PanRHV2 (AF346490); KSHV (U75698, U93872 and AF005477); PanRHV1a (AF250879 and AF250880); PanRHV1b (AF250881 and AF250882); GorRHV1 (AF250886); MndRHV1 (AF282943); MndRHV2 (AF282937 to AF282940); herpesvirus saimiri (HVS) (M31122); ateline herpesvirus 3 (HVA3) (AF083424); Chlorocebus rhadinovirus 1 (ChRV1) (AJ251573); ChRV2 (AJ251574); retroperitoneal fibromatosis–associated herpesvirus strains from *Macaca mulatta* (AF005479) and *M. nemestrina* (AF005478) called here RFHVMm and RFHVMn, respectively; *M. mulatta* rhadinovirus RRV/17577 (AF083501); rhesus monkey rhadinovirus (RRV/H26-95) (AF029302); baboon γ*-*herpesvirus (PapRV2) (AY270026); *Macaca* γ-virus strains from *M. mulatta* (MGVMm) (AF159033), *M. fascicularis* (MGVMf) (AF159032), and *M. nemestrina* (AF159031); and HHV6A (X83413). Other sequences used to construct the phylogenetic trees (especially Epstein-Barr virus and related strains) have been published mainly by Ehlers et al. (*9*) and are named in the legend of Figure 2.

**Figure 2:**
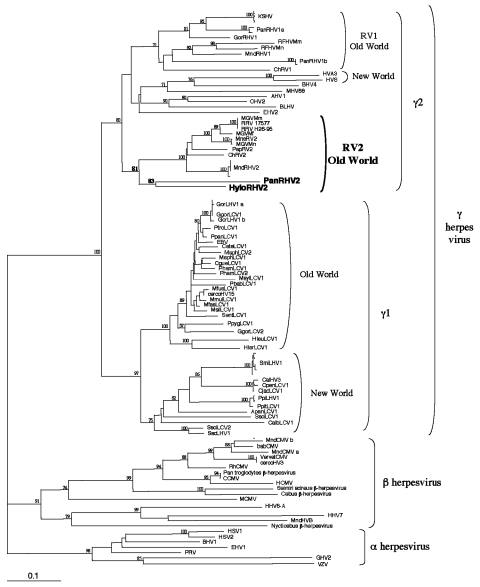
Phylogenetic tree resulting from analysis of selected 351-bp fragments of herpesvirus DNA polymerase gene, which is available for all viruses. The DNA sequences were first aligned by using ClustalX (nonphylogeneticaly informative gaps were manually removed), then the phylogeny was derived by the neighbor-joining method applied to pairwise sequence distances calculated by the Kimura two-parameter method (transition-to-transversion ratio set at 1.15 as expected by a previous Maximum Likelihood analysis). Horizontal branch lengths are drawn to scale, with the bar indicating 0.1 nucleotide replacements per site. Numbers at each node indicate the percentage of bootstrap samples (of 1,000) in which the cluster to the right is supported. Brackets on the right indicate previously defined subfamily and genus herpesviral classification. Previously published sequences and their accession numbers are described online at: http://www.cdc.gov/ncidod/EID/vol10no5/03-0964-G2.htm

The prevalence of HyloRHV2 infection, in our series of gibbons, was determined by Southern blot hybridization of heminested PCR products, with a specific oligonucleotide probe (HyloRHVpr: TTA CGG CTT TAC TGG GGT GGC GAG). Only one sample (gibbon 7) scored positive. Furthermore, we also developed a nested PCR assay using specific primers targeted to the new HyloRHV2 strain (HyloRHVs: GCA TCC CTC CCT GAC AGA GAA TG, HyloRHVas: CAT CGT GCG TCC CTG CAG CG). PCR products hybridization with the specific internal oligonucleotide probe (HyloRHVpr), only detected the same positive sample.

We also tried to specifically amplify an RV2-related virus in our series of DNA samples from orangutans using novel and specific primers targeted to RV2 genogroup strains. None of the 27 DNA scored positive, a result which might be explained either by the absence of an orangutan RV2-related virus in these DNA samples or by its presence at a level not detectable by our method, a situation similar to that observed for KSHV infection in humans. Indeed, in PBMC DNA, PCR analyses detect KSHV viral fragments in only 10% to 20% of KSHV-seropositive healthy persons (*15*).

In conclusion, our data demonstrated for the first time the existence of a gibbon rhadinovirus. Furthermore, after the recent demonstration of two distinct *Rhadinovirus* lineages within the common chimpanzees (*7*), and based on the known molecular evolution of herpesviruses, our findings of a novel RV2 virus in gibbon may suggest the possible existence of a novel γ-2-herpesvirus in humans, belonging to the RV2 genogroup.
